# Nutritional deviations in children: comparative analysis of data from the food and nutrition surveillance system and those obtained by anthropometrists

**DOI:** 10.1590/1984-0462/2022/40/2020439

**Published:** 2021-10-04

**Authors:** Dixis Figueroa Pedraza

**Affiliations:** a Universidade Estadual da Paraíba, Campina Grande, PB, Brazil.

**Keywords:** Anthropometry, Nutrition assessment, Nutritional surveillance, Nutrition programs, Child, Antropometria, Avaliação nutricional, Vigilância nutricional, Programas de nutrição, Criança

## Abstract

**Objective:**

To compare the prevalence rates of nutritional deviations in children under five years of age according to data from the Food and Nutrition Surveillance System (*Sistema de Vigilância Alimentar e Nutricional* — SISVAN) and those obtained by trained anthropometrists.

**Methods:**

This is a descriptive cross-sectional study based on data from 10 municipalities and 2 sources: i) SISVAN Web (secondary database) and ii) an investigation that evaluated the implementation of food and nutrition actions in the Family Health Strategy in the state of Paraíba (primary database), with 24,137 and 897 individuals, respectively. Proportions of overweight — according to weight/age (W/A), weight/height (W/H), and body mass index/age (BMI/A) — and stunting — according to the height/age (H/A) index — based on classifications of SISVAN Web and those obtained by trained anthropometrists were compared using the two-proportion Z-test.

**Results:**

Frequencies of overweight, according to W/A (10.0 vs. 7.8%), W/H (17.2 vs. 14.3%), and BMI/A (18.1 vs. 14.4%), as well as stunting (12.3 vs. 8.6%), were higher on data from SISVAN Web than on those obtained by trained anthropometrists, and the differences were significant.

**Conclusions:**

The findings point to distortions in the nutritional classification of children under five years of age monitored by SISVAN Web.

## RESUMO

**Objetivo:**

Comparar as prevalências de desvios nutricionais em crianças menores de cinco anos de idade de acordo com dados do Sistema de Vigilância Alimentar e Nutricional (SISVAN) com as obtidas por antropometristas treinados.

**Métodos:**

Realizou-se estudo transversal descritivo contemplando dados de 10 municípios procedentes de duas fontes: i) do SISVAN Web (base secundária) e ii) de uma pesquisa de avaliação da implantação das ações de alimentação e nutrição na Estratégia Saúde da Família no estado da Paraíba (base primária); com 24.137 e 897 indivíduos, respectivamente. As proporções de peso elevado, segundo os índices peso/idade (P/I), peso/estatura (P/E) e índice de massa corpórea/idade (IMC/I), e de estatura baixa segundo o índice estatura/idade (E/I), entre as classificações do SISVAN Web e as referidas aos dados obtidos por antropometristas treinados, foram comparadas por meio do teste Z de diferença de proporções.

**Resultados:**

As frequências de peso elevado, de acordo com o P/I (10,0 vs. 7,8%), o P/E (17,2 vs. 14,3%) e o IMC/I (18,1 vs. 14,4%), assim como de estatura baixa (12,3 vs. 8,6%), foram maiores com base nos dados do SISVAN Web do que as obtidas quando foram usadas as medidas realizadas por antropometristas treinados, sendo as diferenças significantes.

**Conclusões:**

Os achados apontam distorções nas classificações nutricionais das crianças menores de cinco anos de idade acompanhadas pelo SISVAN Web.

## INTRODUCTION

Information on the population's nutritional status is a positive health indicator, unlike other morbidity and mortality indicators.^1^ The National Food and Nutrition Policy has the food and nutrition surveillance (*vigilância alimentar e nutricional* — VAN) as one of its guidelines, within a context of expanded focus for surveillance of health services and integration with other information systems.^2^ VAN aims to monitor the nutritional status and food consumption of the population that uses the primary care of the public health system (*Sistema Único de Saúde* — SUS) as a means of formulating and evaluating actions, programs, and policies directed at promoting adequate and healthy eating habits, as well as preventing and treating nutritional diseases.^3^ ^,^ ^4^ In this context, the Food and Nutrition Surveillance System (*Sistema de Vigilância Alimentar e Nutricional* — SISVAN) was implemented as a basic VAN instrument to overcome weaknesses that limit its interface with health and nutrition policies, such as the high cost of population surveys and the low representativeness in nutrition monitoring campaigns.^5^

SISVAN collects, processes, and continuously analyzes data on the food and nutritional status of primary care users. It seeks to contribute to the elaboration of interventions for risk factors associated with nutritional diseases and for social determinants of food and nutrition insecurity, encompassing both individual and collective care.^5^ ^,^ ^6^ When compared to large population-based nutrition surveys, SISVAN data provide information faster, steadily, and at a lower cost. Such information is essential to the formulation and evaluation of food and nutrition interventions.^7^ Also, the system is an important tool for the organization and improvement of nutritional care,^8^ in addition to being the main provider of information on the health of families benefiting from the Bolsa Família Program (a Brazilian welfare program).^1^ The expansion and optimization of SISVAN are among the principal priorities of the SUS food and nutrition area.^8^

Currently, the growth and consolidation of SISVAN face some challenges, such as work fragmentation, lack of structure, and failures in collection standardization, making it difficult to transmit the collected data and ensure its quality, as well as to type and include them in different platforms.^6^ ^,^ ^9^ Thus, evaluating the system, whose literature is still lacking,^10^ is an important tool for assessing its quality and assisting managers in formulating and monitoring food and nutrition policies.^9^ ^,^ ^11^ This study focused on anthropometry rather than on food consumption, as well as on children under five years of age instead of individuals in other stages of life, because SISVAN is more advanced in assessing the nutritional status of the child population.^4^ ^,^ ^6^

Based on the arguments above, the present research aimed to compare the prevalence rates of nutritional deviations in children under five years of age according to data from SISVAN and those obtained by trained anthropometrists.

## METHOD

This is a descriptive cross-sectional study involving children under five years of age treated in primary care units in municipalities from the state of Paraíba in 2017–2018. The research included 10 municipalities (Bayeux, Cabedelo, Cajazeiras, Esperança, Mamanguape, Monteiro, Pombal, Queimadas, São Bento, and Sousa) with populations between 30,000 and 149,999 inhabitants, which receive grants to implement measures to prevent and control overweight in children within the Health at School Program (*Programa Saúde na Escola* — PSE).^12^ Out of the 12 cities with this size receiving the aid, 2 were excluded: the only municipality without full ESF coverage, verified by the percentage of the population covered by ESF teams compared to the population estimate (https://egestorab.saude.gov.br/paginas/acessoPublico/relatorios/relHistoricoCoberturaAB.xhtml, accessed on June 7, 2019) and a city that was considered for PSE implementation analyses.

The information used in this study originated from SISVAN Web and from multifaceted and multistage evaluation research on the implementation of food and nutrition actions in ESF in Paraíba. Two municipalities included in this work had their data collected from the 2017 SISVAN Web public reports, while the others had data from the 2018 reports. Both periods correspond to the data collection intervals of the previously mentioned research.

The representativeness of SISVAN Web data regarding the monitoring of the nutritional status of children under five years of age was confirmed by calculating the system coverage in 2017–2018.^13^ In each municipality, the calculation involved dividing the number of children under 5 years of age monitored by the system (24,137 records according to SISVAN Web data — https://sisaps.saude.gov.br/sisvan/relatoriopublico/index, accessed on March 25, 2020) by the total number of individuals in this age group covered by ESF (38,140 children) and multiplying the result by 100. In the cities investigated, the number of children living in areas covered by family health teams was proportionally estimated based on information about the total population with primary care coverage (available at: https://egestorab.saude.gov.br/paginas/acessoPublico/relatorios/relHistoricoCoberturaAB.xhtml, accessed on March 25, 2020) and the percentage of children under five years of age compared to the total population according to the 2010 Demographic Census (available at: https://censo2010.ibge.gov.br/sinopse/index.php?uf=25&dados=26, accessed on March 25, 2020).

In the analysis research on the implementation of food and nutrition actions in ESF in Paraíba, the study population consisted of children aged 0 to 59 months users of ESF (n=38,140) who lived in the municipalities selected to participate in the investigation. The sample size was calculated considering a two-tailed significance level of 5% (α=0.05), a 95% confidence interval (95%CI), a 90% statistical power (β=0.10), an exposed and non-exposed ratio of 1:1, an expected proportion of the outcome in the non-exposed group of 20% (prevalence of overweight in children, used as an indicator to decide the inclusion of municipalities regarded as a priority for the development of actions aimed at preventing childhood obesity),^12^ and an expected prevalence ratio of 1.5, indicating the need to include at least 790 individuals. An increment of 15% was added to this number to compensate for possible losses and control confounding factors, totaling a sample of 909 children. The Epi-Info software (version 7.2) performed the calculation. The study included 897 children recruited from 46 ESF health teams and 17 daycare centers linked to them through PSE; the sample was proportionally established based on the population aged 0–4 years of each municipality. The selection of health teams, daycare centers, and children was based on a simple random draw.

Children were evaluated at health units or daycare centers. Weight and length (<24 months) or height (≥24 months) were measured following the procedures recommended by the World Health Organization (WHO).^14^ During the entire study, the measurements were obtained by the same anthropometrists, who were properly trained and calibrated. All measurements were taken twice, and the mean value was recorded in a specific form elaborated for this purpose. Children under 24 months had their length measured in the supine position; those aged 25–60 months had their height measured in the orthostatic position. In infants, weight was obtained by the difference between the combined weight of the mother holding the child and the maternal weight. All weights were taken with the individuals wearing light clothes. The equipment used belonged to the research: a wooden anthropometer with a range of 130 cm in 0.1 cm increments, a stadiometer (WCS^®^) with a range of 200 cm in 0.1 cm increments, and a platform-type electronic scale with a capacity of 150 kg and readability of 100 g (Tanita UM-080^®^).

In order to describe the nutritional status, weight/age (W/A), weight/height (W/H), body mass index/age (BMI/A), and height/age (H/A) were calculated in z-scores, based on the WHO growth curves,^14^ using the Anthro software (Centers for Disease Control and Prevention, Atlanta, United States). H/A corresponds to the child's linear growth, including length (<24 months) and height (≥24 months).

Children were classified into underweight, appropriate weight, or overweight categories according to W/A, W/H, and BMI/A, and into stunting or appropriate height groups according to H/A. In SISVAN Web reports, W/A considered very low and low for age were grouped and categorized as underweight. Regarding W/H and BMI/A, pronounced thinness and thinness were grouped and categorized as underweight; normal weight and overweight risk were considered appropriate weight; overweight and obesity were classified as overweight. As to H/A, low and very low height were grouped and categorized as stunting. With respect to the anthropometric data obtained by trained anthropometrists, children with W/A, W/H, and BMI/A lower than or equal to −2 were considered underweight, and those with values greater than or equal to +2 were classified as overweight. Cases with H/A lower than or equal to −2 were regarded as stunting.^14^

The two-proportion Z-test (Z-statistics) compared the proportions of overweight and stunting between classifications made by SISVAN Web and by trained anthropometrists. Differences among the municipalities concerning the distribution behavior of anthropometric results according to data source were also analyzed using the Z-test. For this test, the critical value was set at Z=1.96, considering a 95%CI. A 5% significance level was adopted. Analyses were carried out in the R software, version 2.10.0.

The Research Ethics Committee of the Universidade Estadual da Paraíba approved the research project (opinion No. 2,219,604). Mothers/guardians of the children who participated in the study signed the informed consent form.

## RESULTS

The data used in this study comprised 24,137 cases from SISVAN Web and 897 from the research with primary information. Regarding SISVAN Web data, the distribution of children did not differ according to the city of residence. As for results obtained by trained anthropometrists, the proportion of children in larger cities (Bayeux and Cabedelo) was higher than in the others.

The SISVAN Web coverage for children under 5 years of age in the municipalities investigated was 63.3%. Only 2 municipalities presented variations in the number of SISVAN Web records, with fewer children monitored for W/H (n=24,104) compared to W/A, BMI/A, and H/A records (n=24,137).

Table 1 shows the distribution of the children's nutritional status according to data from SISVAN Web and those obtained by trained anthropometrists. According to SISVAN Web data, overweight frequencies were 10.0, 17.2, and 18.1% for W/A, W/H, and BMI/A, respectively. Also, the stunting incidence was 12.3% among children monitored by SISVAN Web. Lower frequencies of overweight (7.8% for W/A, 14.3% for W/H, and 14.4% for BMI/A) and stunting (8.6%) were identified when using data obtained by trained anthropometrists. Comparing both data sources, the distribution of overweight, in all indices, and stunting presented statistically significant differences. The municipalities showed no differences regarding the distribution behavior of anthropometric results according to data source.

**Table 1:** Distribution of the nutritional status of children under five years of age according to data from the Food and Nutrition Surveillance System and those obtained by trained anthropometrists. Municipalities of Paraíba State, 2017–2018.

Index		Data source				p-value*
		SISVAN		Trained anthropometrists		
		n	%	n	%	
Weight/age						
	Underweight	1,077	4.5	22	2.5	0.019
	Appropriate weight	20,648	85.5	805	89.7	
	Overweight	2,412	10.0	70	7.8	
Weight/height						
	Underweight	1,098	4.7	10	1.1	0.006
	Appropriate weight	18,043	78.1	759	84.6	
	Overweight	3,963	17.2	128	14.3	
BMI/age						
	Underweight	1,308	5.4	13	1.4	<0.001
	Appropriate weight	18,469	76.5	755	84.2	
	Overweight	4,360	18.1	129	14.4	
Height/age						
	Stunting	2,976	12.3	77	8.6	<0.001
	Appropriate height	21,161	87.7	820	91.4	

SISVAN: Sistema de Vigilância Alimentar e Nutricional (Food and Nutrition Surveillance System); BMI: body mass index.

* No difference was found among the municipalities regarding the distribution behavior of anthropometric results according to data source.

## DISCUSSION

The coverage of 63.3% for children under 5 years of age found in the present study is higher than that estimated for this population group in Brazil in 2012 (27.9%)^6^ and for specific locations in the country, such as Rio Grande do Sul in 2010 (10.5%),^13^ municipalities of the Regional Health Superintendency of Belo Horizonte from September to October 2012 (5.6%),^15^ and 2 cities in Paraíba in 2010 (6.5 and 10.1%).^5^ Therefore, the data obtained from SISVAN Web in the municipalities of this study seem to have good representativeness, based on the reported coverage.

The higher coverage found in this research could be expected based on the deliberate choice of municipalities, which should have a SISVAN coverage greater than 10% in 2016.^12^ Other favorable factors can also be mentioned. In Brazil, Paraíba has one of the largest SISVAN Web coverages, possibly influenced by the priority given by certain health and social policies, such as the Bolsa Família Program, to places of greater vulnerability to poverty, hunger, and child undernutrition, as is the case of the Northeast region as a whole.^6^ Also, this study only selected municipalities with full ESF coverage, which can positively influence the system coverage. This finding was confirmed in a national study that showed a positive correlation between ESF and SISVAN Web coverages in 2010, suggesting the importance of this strategy in the expansion, consolidation, and improvement of primary care.^6^ Lastly, it is plausible that the financial incentives received by the municipalities participating in the research also had an influence, as these benefits were directed toward the implementation of actions to prevent and control overweight in children through PSE^12^ and of food and nutrition action in primary care.^16^ Study developed in small municipalities in Rio Grande do Sul on the use of health information systems revealed a relationship between this use and financial transfer.^17^

The prevalence rates of overweight (14.4%) and stunting (8.6%) found in this work are similar to those reported for children under 5 years of age who use public health services, according to a meta-analysis of results from articles published between 2006 and 2014 — 11.0% and 7.3%, respectively.^18^ Other studies based on SISVAN Web data also presented similar results.^5^ ^,^ ^7^ The findings show the coexistence of these two nutritional problems and the nutritional transition process present among Brazilian children,^19^ making the monitoring of nutritional status through SISVAN Web increasingly relevant.^5^ ^,^ ^7^

The Brazilian literature addressing the reliability of SISVAN Web data is still incipient. The results found in the present study corroborate those reported by other researchers, who identified low agreement between nutritional classifications based on SISVAN Web information and on data obtained by trained anthropometrists.^5^ That research, as the present study, detected a higher chance of a diagnosis of impaired linear growth and overweight due to problems in the equipment available in health services and in the techniques used by health professionals.^5^ Similar deficiencies were recorded in the measurement of child body mass by community health agents.^20^ Thus, training professionals in anthropometry is essential to ensure the quality necessary for anthropometric measurements and SISVAN Web information, as previously suggested.^3^ ^,^ ^15^ ^,^ ^20^ ^,^ ^21^ Training should take place once every six months and whenever a change is made in the team of professionals working in the system.^21^

Besides the statistical significance, the differences found between prevalence rates suggest the possibility of misdiagnoses of anthropometric abnormalities among children in health services. This situation has implications for primary care (*atenção primária à saúde* — APS), which should organize nutritional care based on the demands and needs of the territory, considering the main conditions related to food and nutrition. The treatment and care of overweight individuals and those with food problems in Brazil is a challenge for APS, requiring referral to specialized care and possible obstacles in the operation of the health system.^22^ Therefore, primary or specialized care might be directed to individuals who do not need it, restricting adequate care to malnourished children due to the implications of the illness for health services.^22^ ^,^ ^23^

In addition to the characteristics of anthropometric equipment (quality and calibration) and the procedures used by health professionals to obtain anthropometric measurements in their work routine,^5^ ^,^ ^20^ other factors that hinder the SISVAN operationalization may have influenced the differences found in the prevalence rates according to data source. These factors include the lack of control of errors or inconsistencies in data collection and entry.^7^ ^,^ ^8^ ^,^ ^20^ Regarding the population, the importance of the Bolsa Família Program for the use of SISVAN Web should be highlighted, as its public produces a high volume of information for the system, given that the transfer of resources to municipalities depends on the feeding of the SISVAN Bolsa Família.^6^ ^,^ ^7^ Studies have indicated higher prevalence rates of stunting and overweight/obesity among children from families benefiting from this program.^18^ ^,^ ^24^ These families are more vulnerable to food and nutrition insecurity, with possible losses for the child's nutritional status, both in linear growth potential and weight gain.^24^ ^–^ ^27^ The relationship between the findings and the greater use of health services by children with nutritional problems might also be possible.^18^

The results show prevalence rates of overweight varying according to the anthropometric index, especially W/A compared to W/H and BMI/A. This finding can be explained by the applicability of each anthropometric index. While W/H and BMI/A reflect weight and height ratios — adequate to identify overweight —, W/A demonstrates the balance between the child's body mass and chronological age — recommended mainly to know their overall nutritional status and monitor their weight gain.^28^ The use of the W/A index is particularly limited when stunting and overweight coexist, as in this study, indicating apparently normal distributions that may actually represent the combination of nutritional disorders rather than their absence.^7^

The present study was developed in municipalities with full ESF coverage, precluding the generalization of results for the entire state of Paraíba. Moreover, the study did not attempt to understand the main problems faced by health professionals and managers in the operationalization of SISVAN Web. Nonetheless, it warns about the possible overestimation of prevalence rates of overweight and stunting in children under five years of age based on SISVAN data, which could interfere with decisions related to food and nutrition interventions. Thus, the quality of SISVAN Web information is essential for the adequate diagnosis of the population's food and nutritional status and for improving the management of nutrition actions in the ESF. To that end, the use of the theoretical logic model of food and nutritional surveillance in the Brazilian APS is recommended, as it was recently developed and validated by a group of researchers from the country.^3^ ^,^ ^9^ Also, the relevance of the e-SUS Primary Care Strategy in restructuring the methods for collection, processing, validation, and use of health information in APS should be underlined.^29^

The limitations of this study involve the possibility of biases since the SISVAN sample is not random and may include children who are more vulnerable and exposed to conditions related to a worse nutritional status. Furthermore, the non-observation of the measurement procedures restricted the possibilities of explaining the results obtained.

In conclusion, the findings of the present study indicate a lack of reliability of the nutritional data on children under five years of age monitored by SISVAN Web, leading to possible misdiagnoses of stunting and overweight if they are taken into account by food and nutrition management in decision making. The evaluation of children's records makes the subject of special interest to pediatrics since SISVAN makes up strategies for monitoring the food and nutritional status in this life stage and can contribute to defining priorities and allocating resources for interventions in the fields of food and nutrition and of health in the country. Therefore, the need for considering the impacts of this situation on the nutritional care of children and its determining factors should be stressed, aiming at the improvement of food and nutritional surveillance.
